# Conventional versus Reduced-Frequency Follow-Up in Early-Stage Melanoma Survivors: A Systematic Review with Meta-Analysis

**DOI:** 10.3390/curroncol30030256

**Published:** 2023-03-14

**Authors:** Karolina Richter, Tomasz Stefura, Nikola Kłos, Jonasz Tempski, Marta Kołodziej-Rzepa, Michał Kisielewski, Tomasz Wojewoda, Wojciech M. Wysocki

**Affiliations:** 1 Department of Surgery, Faculty of Medicine and Health Sciences, Andrzej Frycz Modrzewski Krakow University, 30-705 Kraków, Poland; karolinaa.richter@gmail.com (K.R.); nikollaklos@gmail.com (N.K.); jonasz.tempski@op.pl (J.T.); mar.2.ta@wp.pl (M.K.-R.); kisialeuskim@gmail.com (M.K.); wojtom67@gmail.com (T.W.); 2 Department of General, Oncological and Vascular Surgery, 5th Military Clinical Hospital in Kraków, 30-901 Kraków, Poland; tomasz.stefura@gmail.com; 3 Department of Medical Education, Jagiellonian University Medical College, 31-008 Kraków, Poland; 4 National Institute of Oncology, Maria Skłodowska-Curie Memorial, 00-001 Warsaw, Poland

**Keywords:** melanoma, follow-up, reduced-frequency, conventional, meta-analysis

## Abstract

To date, there have been multiple studies and clinical guidelines or recommendations for complex management of melanoma patients. The most controversial subjects included the frequency of follow-up. This study provides a coherent and comprehensive comparison of conventional vs. reduced-frequency follow-up strategies for early-stage melanoma patients. The value of our study consists in the precise analysis of a large collection of articles and the selection of the most valuable works in relation to the topic according to rigorous criteria, which allowed for a thorough study of the topic. The search strategy was implemented using multiple databases. The inclusion criteria were randomized clinical trial or cohort studies that compared the outcomes of a conventional follow-up schedule versus a reduced-frequency follow-up schedule for patients diagnosed with melanoma. In this study, authors analyzed recurrence and 3-year survival. Meta-analysis of outcomes presented by Deckers et al. and Moncrieff et. al. did not reveal a significant difference favoring one of the groups (OR 1.14; 95%CI: 0.65–2.00; *p* = 0.64). The meta-analysis of 3-year overall survival included two studies. The statistical analysis showed no significant difference in favor of the conventional follow-up group. (OR 1.10; 95%CI: 0.57–2.11; *p* = 0.79). Our meta-analysis shows that there is no advantage in a conventional follow-up regimen over a reduced-frequency regimen in early-stage melanoma patients.

## 1. Introduction

The number of melanoma cases has drastically increased in recent decades. Not only was there a new record reported in 2022 (325,000 new cases worldwide with mortality of 57,000), but the International Agency of Research on Cancer (IARC) predicted that from 2020 to 2040, melanoma incidence will increase by more than 50%, beyond 500,000 new cases per year, with a simultaneous rise in mortality to almost 100,000 per year [1,2].

Up to this date, there have been multiple studies and clinical guidelines or recommendations for complex management of melanoma patients [1,2]. The most controversial subjects discussed in recent years included the frequency of follow-up. Discussion was fueled by the limited availability of health professionals due to the COVID-19 outbreak. Despite this recent growth in the number of cases of melanoma, which prompted progress in melanoma management, the optimal post-treatment follow-up regimen has remained a controversial topic, with relatively low-quality studies behind current recommendations. This is a particularly significant issue for early melanoma patients, who constitute the majority of the treated population worldwide [3,4]. In this population, the conventional follow-up schedule group (CSG) encompasses frequent patient appointments (every three months through the first year and every four months in the second year, followed by every six months in years 3–5) whereas the reduced-frequency follow-up schedule (experimental follow-up schedule group, ESG) depends on the patient’s state and is individualized, with significantly less frequent clinical appointments compared to CSG [5].

There have been several studies presenting a reduced-frequency follow-up approach for melanoma patients. This approach involves a longer period between follow-up appointments after the essential part of the treatment process. Authors suggested that the conventional approach could burden patients and the health system with too-frequent visits, without significant medical benefit to the patients [6,7,8]. This seems to be particularly important in the age of the COVID-19 pandemic, where every visit to the hospital or outpatient clinic is associated with a risk of cross-infection [9]. A higher frequency of follow-up visits raises the costs of healthcare, when funds might be redirected to more effective fields if lower-frequency follow-ups were proved to be non-inferior [10]. The concept of reduced-frequency follow-ups is of utmost importance, as the global health care is facing limited resources both economically and in terms of manpower.

Therefore, the aim of our study was to provide the most coherent and comprehensive comparison of the follow-up strategies for early stage (T1, T2) melanoma patients. We aimed to sum up the two major approaches (CSG vs. ESG), covering their most important pros and cons.

## 2. Materials and Methods

We registered this study in PROSPERO, receiving a unique identifying number CRD42022366378 [11]. Systematic reviews and meta-analysis do not require ethical approval and the informed consent of the participants.

### 2.1. Search Terms

We conducted research in accordance with the PRISMA guidelines to maintain the highest quality [12]. The PRISMA checklist is available in the supplementary material as File S1 “PRISMA Abstract checklist” and File S2 as “PRISMA checklist” to obtain the highest quality of the study.

The search strategy was implemented using PubMed, Scopus, and Web of Science databases, limited to articles published in English between 1993 and 2022.

Our search strategy was based on the following terms: “melanoma”, “melanomas”, “reduced”, “reduced-frequency” “stage adjusted”, “follow-up”, “follow-up”, “check-up”, “schedule”, “randomized clinical trial”, “cohort”, “clinical-control”, “observational”, and “randomized controlled trial”. Terms were combined with operators such as “AND” and “OR”. 

Authors screened relevant abstracts for inclusion criteria (KR, NK, JT). Senior authors supervised the screening process (TS, MKR, TW, MK, WMW). Selected abstracts were included for full-text analysis in accordance with the below-mentioned inclusion criteria. 

### 2.2. Assessment of Eligibility 

Eligibility assessment for all the full-text articles was performed. The inclusion criterion was a randomized clinical trial or cohort study that compared outcomes of a conventional follow-up schedule with a reduced-frequency follow-up schedule for patients diagnosed with melanoma. The articles must have been written in English. Excluded articles were all meta-analyses, systematic reviews, and any otherwise irrelevant studies. 

### 2.3. Extraction of Data

The data from the included manuscripts were extracted by 8 reviewers. At least two authors extracted data from each article. If any incompatibly of data occurred, the article was reevaluated with a third additional author and one of the senior authors.

### 2.4. Outcomes of Interest

Data were extracted from included studies into an Excel sheet. The database encompassed first author; publication year; title; study design; country of article’s origin; follow-up period in months; sex; median age; sample; study sample divided into groups (CSG and ESG); tumor location: head, trunk, extremities; ulceration; Breslow depth; deaths (for melanoma or other reasons); recurrence; and HR (3-year survival, melanoma specific survival, overall survival, and recurrence free survival). 

### 2.5. Quality Assessment

To evaluate the quality of the included articles, the authors used the Cochrane risk-of-bias tool for randomized trials (RoB 2). The parameters that measured quality were: “Randomization process”, “Deviations from the intended interventions”, “Missing outcome data”, “Measurement of the outcome”, “Selection of the reported result”. Each article was assessed as “high risk”, “low risk”, or “some concerns”. 

### 2.6. Statistical Analysis 

To perform statistical analysis, authors used Review Manager 5.4 (The Cochrane Collaboration, 2020, London, UK). In this study, authors analyzed two parameters: recurrence, 3-year survival. Statistical parameters, such as odds ratios (OR) were generated with 95% confidence intervals (CI), and statistical significance reached 0.05. Each study’s heterogeneity was evaluated by I2 test, and values above 70 constituted considerable heterogeneity.

## 3. Results

### 3.1. Article Selection

**Figure 1:**
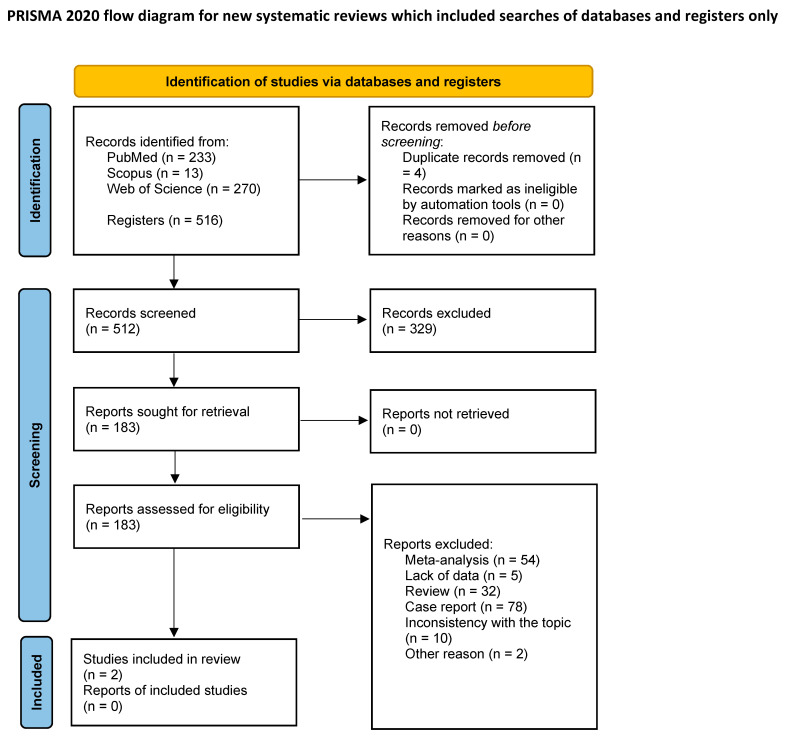
PRISMA flow diagram of the study inclusion process.

The primary search strategy generated 516 records. Finally, 2 full-text articles met the criteria for full-text review, and afterwards, both were included in the meta-analysis [13,14] (Table 1). The remaining 514 records were excluded as per protocol. Details of this process are shown in the PRISMA flow-diagram (Figure 1). 

### 3.2. Characteristics of the Articles

After the record-selection process, two papers from the UK and the Netherlands were included in the statistical analysis. The chosen articles included patients treated between 2006 and 2020 [13,14]. We stratified data into two subgroups to assess the following outcome measures: (1) “Melanoma Recurrence”; (2) “3-year survival”.

### 3.3. Characteristics of the Patients

A total of 387 patients were analyzed. Overall, meta-analysis included 196 patients who underwent a conventional follow-up schedule (CGS) and 191 patients who were included in an experimental reduced-frequency follow-up schedule (ESG). 

### 3.4. Melanoma Recurrence

**Figure 2:**
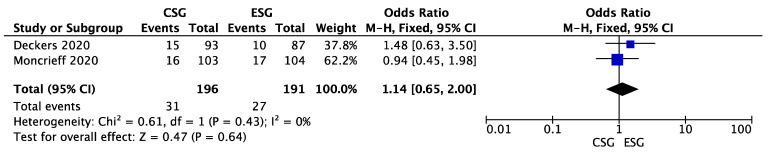
Melanoma recurrence—forest plot [13,14].

A meta-analysis of the studies presented by Deckers et al. and Moncrieff et al. did not reveal a significant difference favoring one of the groups (OR 1.14; 95%CI: 0.65–2.00; *p* = 0.64) [13,14]. The results are presented in Figure 2.

### 3.5. 3-Year Melanoma Survival

**Figure 3:**
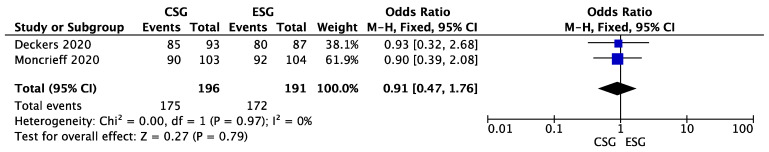
3-year melanoma survival—forest plot [13,14].

The meta-analysis of 3-year overall survival included 2 studies (Deckers et al. and Moncrieff et al.) [13,14]. The statistical analysis showed no significant difference in favor of the conventional follow-up (CSG) group. (OR 0.91; 95%CI: 0.47–1.76; *p* = 0.97). The results are presented in Figure 3. 

### 3.6. Assessment of Quality

The risk for bias in the “Missing outcome data”, “Measurement of the outcome”, and “Selection of the reported result” domains was low. There were concerns regarding the risk of bias in the domains “Randomization process” and “Deviations from the intended interventions”. The quality of the included articles is presented in the supplement as Figure S1.

## 4. Discussion

As melanoma is an increasingly common malignancy, it is very important that patients undergo a methodically planned and stage-tailored posttreatment follow-up schedule for early detection of local, regional, or distant recurrences [15]. The main objective for early detection is rapid introduction of optimal treatment with the potential for long-term benefit [4]. To ensure optimal outcomes, the schedule should be planned in accordance with Evidence-Based Medicine principles. Particularly important is the observation of regional lymph nodes as a potential route for the spread of the cancer, as well as physical examination and imaging studies [16]. 

The conventional approach also has its disadvantages—it can burden patients with too-frequent visits that negatively affect the medical benefit for the patient—especially in the era of the COVID-19 pandemic, which poses a risk of cross-infection [6,7,8,9]. Increasing the frequency of visits may also have a negative impact on hospital facilities, as it increases the cost of medical care, which globally has limited resources both in economic terms and in terms of manpower [10]. Another disadvantage for the patient is over-imaging, which increases the risk of exposure to radiation and overtreating [10].

We aimed to assess whether conventional follow-up was associated with outcomes to those of a reduced-frequency schedule following melanoma therapy. The strengths of our study included scrupulous analysis of the potentially relevant studies initially included, and their integration to allow for clinically meaningful conclusions. 

According to Leiter and Read, there is the highest likelihood of recurrences during the first 3 years after the primary diagnosis. It was shown that up to 50% of recurrences are in the regional lymph node field, 20% are local or in-transit, and up to 30% are distant [7,17,18]. Therefore, it is highly recommended to regularly perform dermoscopic skin examination, educate patients in self-examination, and regularly inspect regional lymph nodes and primary scars every 3–6 months [5,19,20]. 

Our meta-analysis did not present a significant difference favoring the conventional frequency group in terms of melanoma recurrence. Three-year survival was comparable among patients undergoing conventional and reduced-frequency follow-up. Therefore, it seems there is no advantage in subjecting patients with early-stage melanoma (T1, T2) to a conventional frequent follow-up regimen.

According to the results, there is neither a significant difference nor a clinically important benefit in the follow-up schedules compared, although the rigorous follow-up schedule, is more beneficial for regional relapses through self-examination. The great benefit of the reduced follow-up schedule is a major reduction in health care costs compared with the traditional follow-up schedule [18].

There are several limitations precluding the wide implementation of reduced-frequency follow-up schedules. In accordance with most recent European Society of Medical Oncology (ESMO) guidelines, it has not been possible to reach consensus on the optimal scheduling of follow-ups [1,2]. It is beyond question that there is a clinical and psychological value in clinical follow-ups, self-examination, and education of melanoma patients. Our search strategy was strictly planned to include only valid articles directly related to the tested hypothesis, and it consequently generated only two articles related to the topic. Therefore, our study was unbiased by studies only marginally related to our clinical question. Unfortunately, the remaining studies provided no information that was potentially useful for testing our hypothesis, and were excluded. 

Our meta-analysis was associated with several limitations. Unfortunately, the literature on the subject is very limited (we performed a wide literature search resulting in a very small number of valid and directly relevant studies). Moreover, due to the lack of division of melanoma into stages in the included studies, we cannot recommend any follow-up schedule for advanced melanoma. There is a lack of articles including adequate follow-up for T3 and T4 melanoma. Therefore, our results and conclusions are based on a low number of articles and included only T1 and T2 patients. Fortunately, the included papers were high-quality randomized clinical trials, and represented the best available sources of scientifical evidence up-to-date [13,14]. Additionally, T1 and T2 patients constitute the majority of melanoma patients, which makes the results of this meta-analysis highly clinically meaningful and applicable. 

## 5. Conclusions

Our meta-analysis shows that there is no advantage in a conventional follow-up regimen over a reduced-frequency follow-up schedule in early-stage (T1, T2) melanoma patients. A reduced-frequency schedule is optimal, since it reduces unnecessary strain on the health-care system, raises compliance, and—at the same time—does not negatively influence the outcomes. In addition to the recommendation that the reduced-frequency follow-up schedule is non-inferior to the normal follow-up schedule in early melanoma, we speculate that teleconsultations can cover the patient’s psychological need to be contacted frequently by the physician.

## Figures and Tables

**Table 1:** Characteristics of included studies.

Title	The MELFO Study: A Multicenter, Prospective, Randomized Clinical Trial on the Effects of a Reduced Stage-Adjusted Follow-Up Schedule on Cutaneous Melanoma IB–IIC Patients—Results after 3 Years [13]	The MelFo Study UK: Effects of a Reduced-Frequency, Stage-Adjusted Follow-Up Schedule for Cutaneous Melanoma 1B to 2C Patients after 3-Years [14]
Publication year	2020	2020
Study design	RCT	RCT
Country	Netherlands	UK
Sample (n)	180	207
Sex (% of women)	52	47.8
Median age	57	62
Ulceration (n)	25	41
Breslow < 1 mm	8	47
Recurrence total	25	35
HR—3-year disease free	1.24	0.81
HR for overall survival—95% CI	1.06	1.21
HR for recurrence free survival 95% Cl	0.71	1.05

## Data Availability

Not applicable.

## Supplementary Materials

The following supporting information can be downloaded at: https://www.mdpi.com/article/10.3390/curroncol30030256/s1, Figure S1: The quality of the included articles; File S1: PRISMA Abstract checklist; File S2: PRISMA checklist.
