# Supplementary figures and images for: Conventional versus Reduced-Frequency Follow-Up in Early-Stage Melanoma Survivors: A Systematic Review with Meta-Analysis
Source: Curr Oncol. 2023 Mar 14;30(3):3366–72. doi: 10.3390/curroncol30030256 (PMC10046891; doi:10.3390/curroncol30030256)

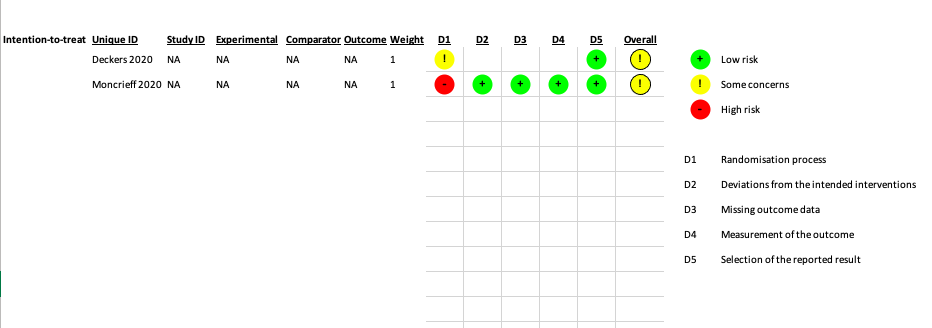

Supplement: Supplementary file 1 [file curroncol-30-00256-s001.zip › Figure S1-The quality of the included articles.png]
